# Israel's repeal of a sweet beverages tax harms public health

**DOI:** 10.3389/fpubh.2023.1231709

**Published:** 2023-12-07

**Authors:** Shelly Kamin-Friedman, Nadav Davidovitch, Y. Tony Yang

**Affiliations:** ^1^Department of Health Policy and Management, Faculty of Health Sciences, School of Public Health, Ben-Gurion University of the Negev, Be'er Sheva, Israel; ^2^Center for Health Policy and Media Engagement, George Washington University, Washington, DC, United States

**Keywords:** sugar-sweetened beverages, taxation, public health, health promotion, obesity, public health policy, health equity, health information

## Background

The consumption of sweet beverages exerts negative consequences on both individual and public health, and their low prices do not reflect the concomitant internalities and externalities (1).

Sweet beverage consumption promotes weight gain in children and adults. A systematic review and meta-analysis of 48 studies in children and 37 studies in adults demonstrates that 1-serving/d greater intake of sugar-sweetened beverages is associated with a 0.07-kg/m^2^ (95% CI: 0.04 kg/m^2^, 0.10 kg/m^2^; *P* < 0.01) higher BMI in children and adolescents and 0.42-kg (95% CI: 0.26 kg, 0.58 kg; *P* < 0.01) higher body weight in adults (2).

Overweight or obesity increase the risk for many serious diseases and health conditions, e.g., hypertension, high levels of triglycerides, type 2 diabetes, coronary heart disease etc. (3). These diseases not only affect health but also cause direct health care costs (4).

The WHO and the World Bank encourage countries to tax sweet beverages to combat obesity and diseases caused by high sugar consumption (5). Studies evaluating these taxes have shown that they lead to increased retail prices, reduced purchases, and a lower consumption of taxed beverages (6). The taxes can also encourage non-price responses such as product reformulation (7).

Although the evidence on the long-term effects of sweet beverage taxes on health outcomes is limited to simulation studies, these studies have consistently demonstrated that applying a sufficiently high tax rate can lead to significant reductions in disability-adjusted life years (DALYs), the prevalence and incident rates of obesity and type 2 diabetes (8).

To date, more than 50 countries outside the United States have already implemented taxes on sweet beverages. However, no US state has taxed these beverages. Some regions and cities have implemented their own local taxes, which are measured in cents per ounce of beverage. The introduction of a 1 cent per fluid ounce sweet beverages tax in Berkeley, California in 2015 resulted in a price increase, a decline in sweet beverage sales, and an increase in untaxed beverages sales, especially water (9). Furthermore, reductions in sweet beverage consumption were observed for at least 3 years and across demographically diverse neighborhoods (10). In contrast, when Cook County, Illinois repealed its sweet beverage tax after 4 months, sales of sweet beverages rebounded to pre-tax levels (11).

## The Israeli experience

On January 1, 2022, a sweet beverages tax went into effect in Israel. It was a purchase tax legislated via the Customs Tariff and Exemptions and Purchase Tax Order on Goods (Amendment No. 4) of 2021.

According to the 2021 amendment, beverages with a sugar content of over 5 grams per 100 ml were taxed at 1 NIS per liter or 6 NIS per liter of concentrate; Sweet beverages with < 5 grams of sugar per 100 mL, sweet beverages containing another sweetener, and fruit juices with more than 5 grams of sugar per 100 mL were taxed at 0.7 NIS per liter of drink or 4.2 NIS per liter of concentrate or powder. “Tirosh” (Israeli kosher grape juice) was exempted from the order, as the high demand for it would not be affected by the tax. The order also exempted lemon juice due to its low sugar content (12).

The Bank of Israel conducted an initial analysis of trends in sweet beverage purchases following the tax's imposition (published on 7 December 2022). A 5% decrease in per capita purchases of sweet beverages was observed alongside a 10% decrease in per capita purchases of high-sugar beverages (13).

However, following the Israeli change of government in November 2022, the new government resolved to repeal the sweet beverage tax in accordance with coalition agreements signed with the Ultra-Orthodox Jewish parties. In January 2023, the Israeli Finance Minister repealed the tax after almost no consultation with civil service professionals in the ministries of health or finance. Both ministries' experts were against repealing the tax as cited unofficially in the media.

The repeal of the tax was met with pushback from the international public health community, as it went counter to clear guidance from such international agencies as the WHO and the World Bank (14).

## The main reasons for the opposition to the sweet beverages tax in Israel

Opposition to sweet beverage taxation generally comes from manufacturers and lobbyists, who seek to protect their profit-making model by resisting efforts to shift drinking patterns toward healthier alternatives (15–18).

The Israeli case, however, also included strong opposition from the Ultra-Orthodox community.

The Ultra-Orthodox community in Israel is a low percentile socioeconomic minority that functions in self-imposed cultural insularity. This population represents 13% of Israel's total population (19) and its health patterns differ from the rest of Israel, with higher rates of obesity and diabetes (20).

An increased consumption of sweet beverages (as well as the consumption of other unhealthy foods) is prevalent in the Ultra-Orthodox community and an inherent part of its culture. A common practice is to give sweets at home and in schools as a reward in order to create motivation for action. Sweets are given to young children in order to make them like the “Torah” and follow its directives. Moreover, the high number of children in Ultra-Orthodox families leads to many celebrations where it is customary to consume sweets (21).

The culture of sweet consumption stems partially from the fact that the Ultra-Orthodox Jews live in tightly-knit communities with limited exposure to secular media (television, newspapers, radio, Internet). They are therefore not exposed to major health information sources (22). The high consumption of sweet beverages is also a specific result of aggressive advertising by the sweet beverage companies in media outlets which target the Ultra-Orthodox community. A study found that in 2020, the number of advertisements on behalf of beverage companies in the Ultra-Orthodox press was 385% higher than the number of advertisements in newspapers intended for the general population (23).

Given the high consumption of sweet beverages in the Ultra-Orthodox community, and the relatively low socioeconomic status of most community members, the taxation of sweet beverages was perceived by the community as an economic burden rather than a tool for promoting health (24). The lack of policy makers' efforts to increase the Ultra-Orthodox community's collaboration and cooperation with a transition to healthier beverages intensified this perception.

Moreover, the tax was also perceived as a political decision that must be repealed since it was imposed by the previous government. The previous government, which did not include representatives from the Ultra-Orthodox sector, made several decisions that disproportionately affected the Ultra-Orthodox community, including taxing disposable utensils, that the Ultra-Orthodox consume heavily, and reforming the granting of “kosherness” [*kashrut*] certificates. Taxing sweet beverages was thus framed as one of many anti-Orthodox policies (25). In the new government, Ultra-Orthodox parties enjoyed significant influence and promised their voters an immediate cancellation of the previous government's decisions, including the taxation of sweet beverages. Political reasons motivated the promise, which was given without consulting with experts.

## Tax cancellation's consequences

The repeal of Israel's sweet beverage tax may lead to a higher consumption of such drinks, potentially causing adverse health effects. Moreover, canceling the tax for political reasons and without consulting health experts diminishes the role of scientific expertise in shaping public health policies.

## Lessons

While implementing a tax on sweet beverages and in addition to dealing with manufacturer opposition, policymakers must demonstrate that it is not an economic sanction or an attempt to target specific communities but a means to promote health.

To achieve this end, policy makers must inform the public about the harms of sweet beverages before imposing the tax. The information should be adapted to different sectors and conveyed through various means. The Ultra-Orthodox community in Israel, for example, requires information through street ads rather than secular media.

Moreover, tax revenues should be earmarked for interventions aimed primarily at promoting health (26). The earmarking of tax revenues for health promotion programs designed for communities with high sweet beverage consumption will enhance community members' confidence that policymakers are acting in their best interests (27).

It is also imperative that sweet beverage taxation be accompanied by a plan to change the sweet beverage consumption habits of the public at large and of closed communities in particular. While planning to reduce sweet beverage consumption in Israel's Ultra-Orthodox community, it was necessary to collaborate with the community representatives, including Ultra-Orthodox health experts and Ultra-Orthodox media. Moreover, and as religious leaders play a significant role in shaping community members' perceptions, policy makers should have taken steps to obtain the support of religious leaders from all Ultra-Orthodox subgroups. Strategies adapted to the customs and cultures of the community could thus have been developed by working together with community leaders and representatives (28, 29).

The judicial review of government decisions is also essential to preventing the repeal of the tax based on irrelevant political motives (30, 31). Courts must thus have the authority to invalidate governmental decisions that harm public health (32, 33).

## Conclusion

The decision to repeal the sweet beverage tax in Israel may lead to a rise in the consumption of these drinks, potentially resulting in health issues like obesity and diabetes. Such a move could also diminish the credibility of health experts and highlight political influence in public health decisions. Israel's situation thus underscores the necessity for public backing before implementing such taxes. Policymakers should ensure these initiatives are viewed as health-driven, not merely economic sanctions. Accordingly, they should actively engage communities in understanding and modifying their beverage consumption habits. Moreover, emphasizing evidence-based policies and community engagement can foster better public health outcomes, rather than allowing narrow political interests to dictate decisions.

## Author contributions

SK-F, ND, and TY were equally involved in drafting this article and approved it for publication. All authors contributed to the article and approved the submitted version.
